# Primary high-grade mucoepidermoid carcinoma of the lacrimal gland: retrospective clinical-pathologic analysis of 20 cases

**DOI:** 10.1186/s12886-026-04784-y

**Published:** 2026-04-17

**Authors:** Xushuang Zhi, Jing Li, Wei Fang, Yueyue Li, Rui Ma, Yan Hei, Xuetao Mu, Yue Li, Lan Yao, Wenlu Liu, Yanchen Liu, Aijun Deng, Jianmin Ma, Xiaohui Lv, Xinji Yang, Wei Wu

**Affiliations:** 1 Senior Department of Ophthalmology, 3rd Medical Center of Chinese PLA General Hospital, Beijing, 100039 China; 2 Beijing Institute of Ophthalmology, Beijing Tongren Eye Center, Beijing Tongren Hospital, Capital Medical University, Beijing, 100730 China; 3 National Clinical Research Center for Ocular Diseases, Eye Hospital, Wenzhou Medical University, Wenzhou, 325027 China; 4 Department of Radiology, 3rd Medical Center of Chinese PLA General Hospital, Beijing, 100039 China; 5 Department of Ophthalmology, Affiliated Hospital of Shandong Second Medical University, Weifang, Shandong 261041 China

**Keywords:** Mucoepidermoid carcinoma, Lacrimal gland, Diagnostic characteristics, Treatment characteristics, Mutational landscape

## Abstract

**Background:**

Primary high-grade lacrimal gland (LG) mucoepidermoid carcinoma (MEC) is a rare condition that presents diagnostic and treatment challenges. In this study, we aimed to elucidate the diagnosis, treatment, and mutational landscape of high-grade LG MEC.

**Methods:**

We reviewed clinical symptoms, radiological images, treatment, prognosis, histopathology, and mutational landscape of 20 patients with high-grade LG MEC.

**Results:**

Primary clinical presentation of high-grade LG MEC was proptosis (85%) with a low incidence of ocular pain (20%). CT scans showed frequent bone destruction (89.5%) and calcification (73.7%). MRI revealed hypointense areas on T2-weighted imaging (WI) in 85% of lesions and non-enhanced areas on contrast-enhanced T1WI in 95% of cases. All 20 patients with primary high-grade LG MEC underwent surgery and were stratified into 5 treatment groups. Multi-group analysis showed only the complete resection plus adjuvant radiotherapy group had significantly better RFS than the complete resection alone group (both *P* = 0.0016), with no significant OS differences across groups. The rates of recurrence, metastasis, and mortality for patients in T4 stage were 77.8%, 71.4%, and 100%, respectively, while those in stages T1-3 were 25%, 75%, and 62.5%. Log-rank tests demonstrated superior long-term RFS and OS in T1–3 patients (*P* < 0.05), whereas Gehan-Breslow-Wilcoxon tests yielded non-significant results. Whole-exome sequencing of tumors and adjacent normal tissues from 3 patients showed TP53 as the only consistent driver gene with missense mutations in all samples. Additionally, EGFR, DOCK2, GAN11, AKAP9, and SMC4 were identified as driver genes in 2 patients.

**Conclusions:**

High-grade LG MEC predominantly manifested as painless exophthalmos and the characteristic imaging included calcification, bone destruction, hypointense areas on T2WI, and non-enhanced areas on contrast-enhanced T1WI. Adjuvant radiotherapy after complete resection reduced recurrence and improved RFS but did not significantly elevate the overall survival rate of primary high-grade LG MEC patients. Prognosis is especially poor for T4 stage. Potential somatic variants identified within this small exploratory cohort, including TP53, EGFR, DOCK2, GAN11, AKAP9, and SMC4 may represent the potential therapeutic targets. Nevertheless, their clinical and prognostic relevance requires rigorous validation in larger independent patient cohorts.

**Supplementary Information:**

The online version contains supplementary material available at 10.1186/s12886-026-04784-y.

## Background

Mucoepidermoid carcinoma (MEC) primarily occurs in the salivary glands and is rarely found in the lacrimal gland (LG). According to the Brandwein Schemes for Grading of MEC, the tumor is classified into low, medium, and high-grade types [1]. High-grade MECs are characterized by poor differentiation, predominantly consisting of squamous epithelial cells, intermediate cells, and less than 10% mucinous cells, resulting in a more aggressive malignancy [2. Due to the extremely low incidence rate of primary high-grade LG MEC, only 21 cases have been documented thus far. The primary high-grade LG MEC is considered to be high malignancy, strong invasiveness and poor prognosis [2–5]. However, the majority of existing studies are case reports, and there is a lack of systematic and comprehensive research, hindering the development of effective diagnostic and therapeutic strategies for primary high-grade LG MEC.

In clinical practice, the preoperative diagnosis of primary high-grade LG MEC is often confounded with primary LG adenoid cystic carcinoma (ACC), which is the most prevalent malignant tumor of the LG. Distinguishing the clinical and imaging features of these two diseases is vital for accurate preoperative identification of primary high-grade LG MEC. Despite the abundance of literature on the clinical and imaging characteristics of primary LG ACC [6, 7] there is a notable scarcity of comparative studies between primary high-grade LG MEC and LG ACC.

In this study, we reported the clinical presentations, imaging characteristics, treatment modalities, prognosis, pathological features, and mutational landscape of 20 patients diagnosed with primary high-grade LG MEC, contrasting these findings with cases of medium-grade LG MEC and LG ACC. We thus presented essential diagnostic and therapeutic considerations for primary high-grade LG MEC. The study represents the largest documented series and the most comprehensive investigation on primary high-grade LG MEC thus far.

## Methods

### Cohort

The cohort included 19 patients with high-grade LG MEC who underwent surgical treatment at the 3rd Medical Center of the Chinese PLA General Hospital between January 2010 and December 2023. Additionally, 16 patients with LG ACC treated surgically at the same institution from January 2013 to December 2023 were included. Two patients with medium-grade LG MEC and 19 with LG ACC, who underwent surgery at Beijing Tongren Hospital between January 2016 and December 2023, were also part of the study. Furthermore, a patient with high-grade LG MEC who underwent surgery at the Eye Hospital of Wenzhou Medical University in November 2023 was enrolled. All participants were primary cases and had been pathologically confirmed.

### Clinical data

Data collection involved reviewing electronic medical records and radiological images. Radiological assessments were conducted by 3 evaluators (X.M., X.Y., and W.W.) using a majority rule approach. Follow-up information was obtained through telephone interviews, outpatient visits, or additional medical records.

### Histology

The paraffin-embedded specimens were sectioned into slices with a thickness of 5 μm for histopathological analysis. Hematoxylin-eosin (HE) staining was employed to assess the morphological features and distribution patterns of tumor cells, whereas alcian blue-periodic acid-schiff (AB-PAS) staining was utilized to identify the presence and distribution of mucus within the samples. Immunohistochemical analysis was conducted using antibodies against CD34 (ZM-0046, ZSGB-Bio, China), EGFR (ZM-0093, ZSGB-Bio, China) and HER2 (ZM-0065, ZSGB-Bio, China).

### Whole-exome sequencing

The pathologically confirmed tumor and the adjacent normal tissue were sectioned into slices with a thickness of 8 μm. DNA samples were extracted from the slices using standard methodologies, ensuring that the genomic DNA samples meet the required criteria for concentration and integrity prior to library construction and sequencing. Genomic DNA from tumor and normal samples were fragmented and used for whole exome library construction and captured using the Agilent V6 exome panel per the manufacturers’ instructions. Paired-end sequencing, resulting in 100 bases from each end of the fragments, was performed using a MGISEQ-2000 Genome Analyzer. Utilizing standard bioinformatics analyses, we identified gene mutations present in tumor and normal tissue samples. By excluding germline mutations found in the matched normal tissues, we isolated the somatic mutations specific to the tumor tissues. Subsequently, these somatic mutations were compared against established driver genes documented in databases (pan-cancer and Cancer Gene Census) and 4 widely recognized papers to identify potential driver genes within the tumor samples [8–11]. High frequency mutation genes take into account the single nucleotide variation (SNV) and insertion and deletion (InDel) mutations in somatic cells, which means that the mutation frequency is significantly higher than the background mutation frequency. MuSic software was used to analyze high frequency mutation of tumor.

### Statistical analysis

For statistical analysis in all primary LG MEC, continuous variables following a normal distribution were expressed as mean ± standard deviation (SD), while those not normally distributed were presented as median (25th percentile, 75th percentile). Statistical analyses were carried out using SPSS software, and a p-value of less than 0.05 was considered statistically significant. A total of 20 patients with primary high-grade LG MEC were enrolled, all of whom received surgical treatment and were stratified into five groups according to treatment modalities (complete resection with adjuvant radiotherapy, complete resection alone, etc.). Kaplan-Meier method was used to construct survival curves, and Log-rank (Mantel-Cox) test, Gehan-Breslow-Wilcoxon test and Bonferroni correction were performed to analyze the differences in recurrence-free survival (RFS) and overall survival (OS) among groups. The effects of tumor TNM staging and patient age on prognosis were further explored.

## Results

### The prevalent population and clinical presentations of primary high-grade LG MEC differed from those of LG ACC

We reviewed the clinical data of 20 patients with primary high-grade LG MEC and found that their average age was 60.5 ± 13.6 years (range: 32–84), with a male to female ratio of 17:3. All cases were unilateral. The most prevalent clinical symptoms included proptosis (85%), ocular dyskinesia (75%), palpable periorbital mass (65%), eyeball displacement (60%), eyelid swelling (60%), and ptosis (30%). Notably, visual loss and ocular pain were less frequently reported, each occurring in only 20% of cases.

Two patients with medium-grade LG MEC both presented with painless exophthalmos, ocular dyskinesia, palpable periorbital mass, and no visual impairment. Obviously, the clinical manifestations between high and medium grade LG MECs showed no significant differences. Conversely, data from 35 patients with LG ACC revealed significant demographic and symptomatic differences. The average age of primary high-grade LG MEC patients was significantly higher compared to LG ACC patients, who had an average age of 41.2 ± 15.6 years. The male-to-female ratio in LG ACC was more balanced at 17:18, markedly different from the male predominance observed in high-grade LG MEC. Furthermore, the incidence of ocular pain differed significantly between the groups, with only 20% in high-grade LG MEC versus 54.3% in LG ACC (*p* = 0.022), as detailed in Supplementary Table S1.

In summary, primary high-grade LG MEC predominantly affects older males and is primarily characterized by progressive painless exophthalmos. Compared to LG ACC, notable distinctions include an older average age at onset, a higher male predominance, and a significantly lower occurrence of ocular pain in patients with high-grade LG MEC.

### The calcification patterns and MRI appearances on T2WI and contrast-enhanced T1WI were distinctly characteristic in primary high-grade LG MEC compared to LG ACC

**Fig. 1:**
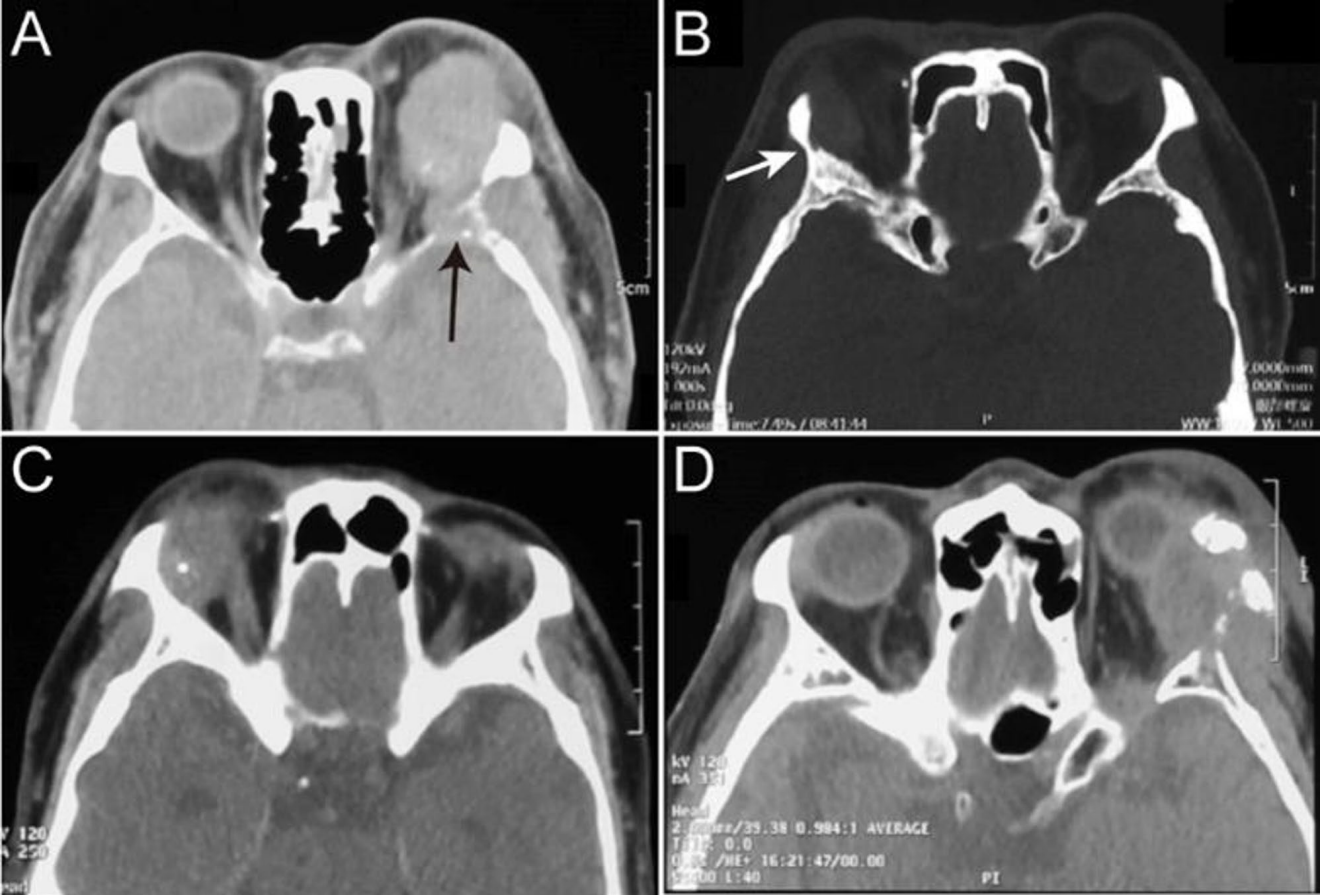
CT characteristics of primary high-grade LG MEC. (**A**) CT scan illustrating a primary high-grade LG MEC lesion with pronounced bone destruction, as indicated by the arrow. (**B**) CT scan demonstrating a primary high-grade LG MEC lesion with subtle bone destruction, highlighted by the arrow. (**C**) CT scan showing a primary high-grade LG MEC lesion characterized by punctate calcification. (**D**) CT scan depicting a primary high-grade LG MEC lesion with extensive calcification and significant bone destruction

We obtained preoperative 19 computed tomography (CT) and 20 magnetic resonance imaging (MRI) scans from the patients with primary high-grade LG MEC. The imaging revealed irregular and poorly defined LG lesions in close proximity to the extraocular muscles. CT scans revealed that the lesions consistently exhibited soft tissue density (Fig. 1A-D), with 89.5% (17/19) displaying relatively uniform density. High rates of bone destruction were observed in 89.5% (17/19) of cases (Fig. 1A), with 31.6% (6/19) requiring careful review due to subtle bone destruction (Fig. 1B). Another notable characteristic feature in primary high-grade LG MEC was a high prevalence of calcification at 73.7% (14/19), primarily manifesting as punctate calcifications in 85.7% (12/14) of cases (Fig. 1C), with only 2 instances of large calcific deposits (Fig. 1D).

**Fig. 2:**
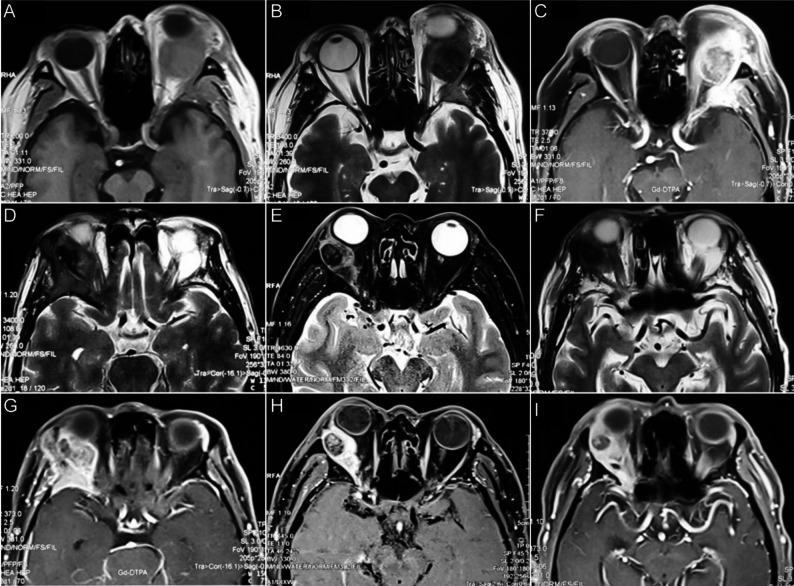
MRI characteristics of primary high-grade LG MEC. (**A**) T1WI showing the primary high-grade LG MEC lesion as isointense. (**B**) T2WI displaying the primary high-grade LG MEC lesion as isointense with a hypointense area. (**C**) Contrast-enhanced T1WI illustrating peripheral enhancement with a central non-enhanced areas in the primary high-grade LG MEC lesion. MRI scans in panels **A**-**C** are from the same patient. (**D**-**F**) T2WI from different patients showing primary high-grade LG MEC lesions characterized by isointense with hypointense areas. (**G**-**I**) Contrast-enhanced T1WI from different patients showing primary high-grade LG MEC lesions characterized by non-enhanced areas. MRI scans in **D** and **G**, **E** and **H**, and **F** and **I** are from the same patients

MRI scans revealed that lesions typically appeared isointense on T1-weighted images (WI) (20/20, 100%) (Fig. 2A), isointense on T2WI (16/20, 80%) (Fig. 2B) and exhibited marked enhancement on contrast-enhanced T1WI (17/20, 85%) (Fig. 2C). Of particular interest was the presence of hypointense areas in 85% of lesions on T2WI (Fig. 2B, D-F), and the presence of non-enhanced areas in contrast-enhanced T1WI in 95% (19/20) of cases (Fig. 2C, G-I).

In contrast, the 2 cases of medium-grade LG MEC exhibited more regular, quasi-circular lesions with clearer boundaries. Preoperative CT imaging also revealed bone destruction and punctate calcifications, with lesions demonstrating isointense on T1WI but exhibiting noticeable variations on T2WI and enhanced sequences. Specifically, one case displayed isointense on T2WI and prominent enhancement on contrast-enhanced T1WI interspersed with non-enhanced areas (Supplementary Figure S1A-C), while the other exhibited hyperintensity on T2WI and no enhancement on contrast-enhanced T1WI (Supplementary Figure S1D-F). In comparison to LG ACC, primary high-grade LG MEC was more likely to exhibit calcifications on CT, more frequently appeared hypointense on T2WI, and exhibited non-enhanced areas on contrast-enhanced T1WI (Supplementary Table S2).

### Complete resection received adjuvant radiotherapy demonstrated limited therapeutic effect for primary high-grade LG MEC

All the 20 patients with primary high-grade LG MEC underwent surgical treatment, 11 patients who underwent complete resection received adjuvant radiotherapy, 4 patients who underwent postoperative radiotherapy for gross residual disease, 2 patients underwent adjuvant chemotherapy, and 1 patient was treated with targeted therapy (lenvatinib) (Supplementary Table S3). Over a follow-up period of 35.5 months (19.8, 62.3), the observed recurrence rate among these 20 primary high-grade LG MEC patients was 52.9% and the metastasis rate was 71.4%. The lungs were the most common site of distant metastasis (63.6%), followed by the salivary glands and bones (27.3%), with occasional occurrences of brain metastases (9.1%). Shockingly, the mortality rate was as high as 85%, with a 5-year mortality rate of 65%. Compared to this, patients with medium-grade LG MEC had a better prognosis, experiencing no recurrence, metastasis, or mortality during follow-up periods of 10 and 59 months.

**Fig. 3:**
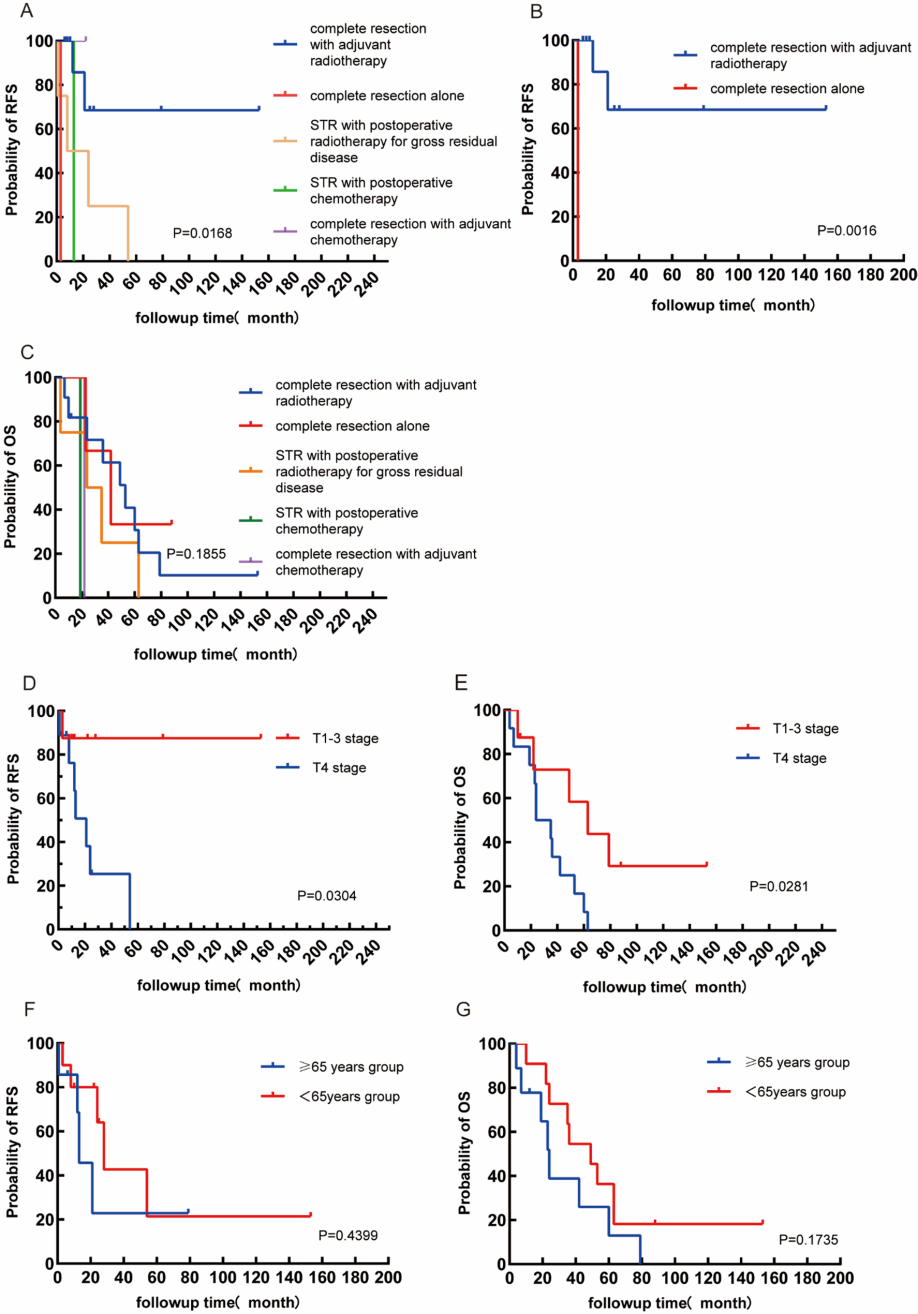
Kaplan-Meier survival curves for patients with primary high-grade LG MEC. (**A**) Recurrence-free survival (RFS) by treatment modality (Log-rank *P* = 0.0168). (**B**) RFS comparing complete resection with adjuvant radiotherapy vs. complete resection alone (Log-rank *P* = 0.0016). (**C**) Overall survival (OS) by treatment modality (Log-rank *P* = 0.1855). (**D**) RFS stratified by T stage (T1-3 vs. T4, Log-rank *P* = 0.0304). (**E**) OS stratified by T stage (T1-3 vs. T4, Log-rank *P* = 0.0281). (**F**) RFS stratified by age (≥ 65 vs. <65 years, Log-rank *P* = 0.4399). (**G**) OS stratified by age (≥ 65 vs. <65 years, Log-rank *P* = 0.1735). Abbreviations: RFS, recurrence-free survival; OS, overall survival; STR, subtotal resection

Surgical records indicated that 15 out of 20 patients achieved complete surgical resection. However, the remaining 5 patients underwent subtotal excision due to the ill-defined nature of the tumors and their proximity to critical tissues. Patients were divided into five groups according to treatment modality: complete resection with adjuvant radiotherapy, complete resection alone, complete resection with adjuvant chemotherapy, subtotal resection (STR) with postoperative radiotherapy for gross residual disease, and STR with postoperative chemotherapy, our analysis concentrated predominantly on the impact of treatment. Multi-group survival analysis revealed significant differences in Recurrence-free survival analysis (RFS) curves (Fig. 3A) among the five groups (Log-rank: χ²=12.08, df = 4, *P* = 0.0168; Gehan-Breslow-Wilcoxon: χ²=10.48, df = 4, *P* = 0.0331). Pairwise Log-rank tests were then performed with Bonferroni correction for multiple comparisons (adjusted α’=0.005), and only the comparison between the complete resection with adjuvant radiotherapy group and the complete resection alone group remained statistically significant (Fig. 3B) (Log-rank χ²=10.00, df = 1, *P* = 0.0016;Gehan-Breslow-Wilcoxon χ²=10.00, df = 1, *P* = 0.0016), with no significant differences observed in other pairwise comparisons. For Overall survival analysis (OS), neither the Log-rank (Mantel-Cox) test (χ²=6.189, df = 4, *P* = 0.1855) nor the Gehan-Breslow-Wilcoxon test (χ²=4.497, df = 4, *P* = 0.3429) detected significant differences in survival curves across the five groups (Fig. 3C). The Log-rank test for trend was also not significant (χ²=3.088, df = 1, *P* = 0.0789), indicating no significant linear gradient in OS across the groups. Collectively, these findings demonstrate that treatment modality grouping is not significantly associated with overall survival prognosis in this cohort. These findings indicated that adjuvant radiotherapy following complete resection can reduce recurrence risk and is essential for improving recurrence‑free survival, it does not substantially enhance survival rate for primary high-grade LG MEC.

### T4 primary high-grade LG MEC exhibited the worst prognosis

We further investigated the influence of tumor TNM staging and patient age on prognosis. We found that patients with T4 primary high-grade LG MEC exhibited recurrence, metastasis, and mortality rates of 77.8%, 71.4%, and 100%, respectively, in contrast to rates of 25%, 75%, and 62.5% for stages T1-3 (Table 1). Kaplan-Meier survival curves were constructed, and Log-rank (Mantel-Cox) and Gehan-Breslow-Wilcoxon tests were performed to compare survival outcomes between patients with T1–3 stage and T4 stage disease. Consistent results were observed in two independent analyses: the Log-rank test revealed significant differences in survival curves between the two groups for both recurrence-free survival (RFS) (Fig. 3D) and overall survival (OS) (Fig. 3E) (RFS: χ² = 4.689, df = 1, *P* = 0.0304; OS: χ² = 4.822, df = 1, *P* = 0.0281). In contrast, the Gehan-Breslow-Wilcoxon test did not reach statistical significance for either endpoint (RFS: χ² = 2.385, df = 1, *P* = 0.1225; OS: χ² = 2.745, df = 1, *P* = 0.0975). These findings indicated that the prognostic impact of T stage is primarily evident in long-term follow-up, with no significant early-stage differences between groups. Median survival analysis confirmed a significantly better prognosis in T1–3 stage patients: the median survival was undefined for T1–3 stage patients and 21.00 months for T4 stage patients in the RFS analysis; the median survival was 63.00 months for T1–3 stage patients and 29.50 months for T4 stage patients in the OS analysis, with a median survival ratio of 2.136 (95% CI: 0.7524 to 6.062). Collectively, these results demonstrated that T4 stage is a critical risk factor associated with significantly poorer long-term survival. However, due to the limited sample size, the 95% confidence interval of the median survival ratio includes 1, indicating statistical uncertainty that requires validation in larger cohort studies.

Furthermore, individuals aged 65 and older were observed to have a higher likelihood of recurrence, although there were no significant disparities in metastasis and mortality rates between different age cohorts (Table 1). Kaplan-Meier survival curves were constructed, and Log-rank (Mantel-Cox) and Gehan-Breslow-Wilcoxon tests were performed to compare OS and RFS between the two groups (Fig. 3F and G). Both the Log-rank (Mantel–Cox) and Gehan–Breslow–Wilcoxon tests revealed no significant differences in overall survival curves between patients aged ≥ 65 years and those aged < 65 years (Log-rank: χ² = 0.5967, df = 1, *P* = 0.4399; Gehan–Breslow–Wilcoxon: χ² = 0.8000, df = 1, *P* = 0.3711). The median overall survival was 13.00 months in the older group and 28.00 months in the younger group, with a median survival ratio of 0.4643 (95% CI: 0.1247–1.729). Since the confidence interval crossed unity, these data further confirmed the absence of statistically significant survival differences, indicating that age stratification is not an independent prognostic factor for overall survival in this cohort.

**Table 1:** Prognosis of primary high-grade LG MEC

Prognostic Factor	Recurrence (*n* = 17)	Metastasis (*n* = 15)	Mortality (*n* = 20)
Primary high-grade LG MEC overall	52.9% (9/17)	71.4% (11/15)	85% (17/20)
Complete excision	33.3% (4/12)	81.8% (9/11)	80% (12/15)
Subtotal excision	100% (5/5)	50.0% (2/4)	100% (5/5)
T4 stage	77.8% (7/9)	71.4% (5/7)	100% (12/12)
T1-3 stage	25% (2/8)	75% (6/8)	62.5% (5/8)
Aged ≥ 65 years	80% (4/5)	80% (4/5)	88.9% (8/9)
Aged < 65 years	41.7% (5/12)	70% (7/10)	81.8% (9/11)

### Primary high-grade LG MEC was prone to necrosis and invade nerve and bone tissue

**Fig. 4:**
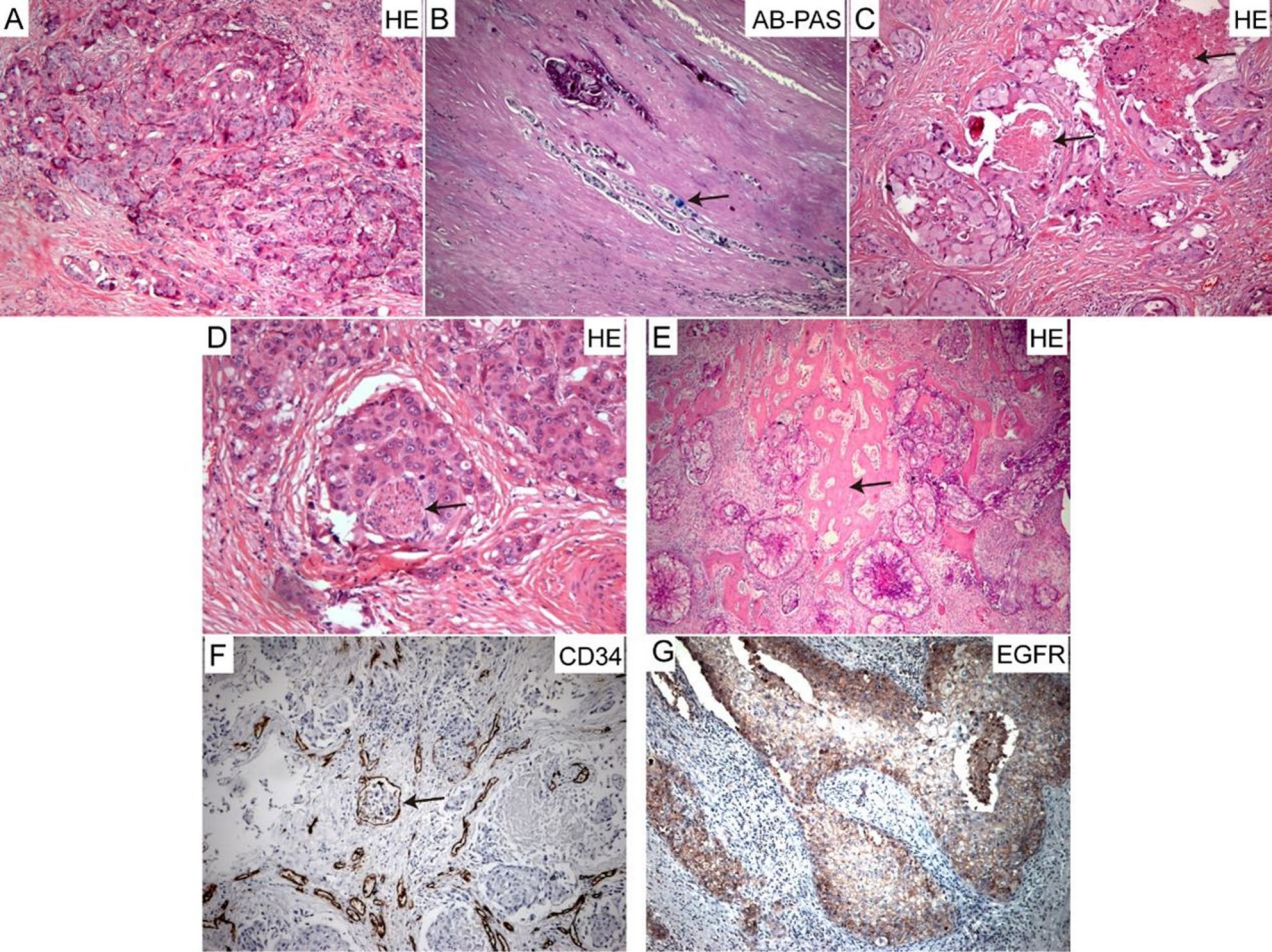
Histopathological examination of primary high-grade LG MEC. (**A**) H&E staining illustrated that the primary high-grade LG MEC tumor was predominantly composed of epidermoid cells. (**B**) AB-PAS staining revealed that the tumor stroma of the primary high-grade LG MEC was rich in collagen, with rare mucus presence, as indicated by the arrow. (**C**) H&E staining displayed coagulative necrosis within the primary high-grade LG MEC lesion. (**D**) H&E staining showed that the primary high-grade LG MEC tumor invaded neural tissue, as indicated by the arrow. (**E**) H&E staining demonstrated invasion of bone tissue by the primary high-grade LG MEC tumor, highlighted by the arrow. (**F**) Immunohistochemistry revealed CD34 + blood vessel invasion by the primary high-grade LG MEC tumor, as marked by the arrow. (**G**) Immunohistochemistry showed that the primary high-grade LG MEC tumor was positive for EGFR

Analysis of the histological characteristics of primary high-grade LG MEC tumor specimens revealed that the tumor cells predominantly consisted of epidermoid cells with few mucous cells (Fig. 4A). The tumor stroma was rich in collagen but contained little mucous (Fig. 4B). Additionally, coagulative necrosis was frequently observed (90%) (Fig. 4C), with common invasion into nerves (55%) (Fig. 4D) and bone tissue (50%) (Fig. 4E), as well as vascular invasion (25%) (Fig. 4F). Notably, the positive rates of ERFR in primary high-grade LG MEC tumor were 63.2% (Fig. 4G). In contrast, 2 cases of medium-grade primary LG MEC did not exhibit necrosis or invasion into nerves, bone, or blood vessels.

### TP53 is the potential driver gene of primary high-grade LG MEC

Whole-exome sequencing was performed on tumour tissues and matched adjacent normal tissues from only three patients in this study. Due to the limited number of successfully profiled samples, these genomic findings remain preliminary and require further validation in larger independent cohorts. We conducted whole-exome sequencing on high-grade LG MEC, utilizing adjacent normal tissue as a control. Due to partial DNA degradation in the samples, we successfully obtained the results of primary cancers with matched normal tissue from 3 patients. We performed a comparative analysis of somatic mutations in the tumors of the 3 patients, aligning our findings with established driver genes documented in databases and literatures, to identify potential driver genes associated with high-grade LG MEC. Our study identified a total of 104 driver genes (Supplementary Table S4), with TP53 being the only driver gene consistently present across all 3 patient specimens, where it exhibited a missense mutation. Additionally, the genes EGFR, DOCK2, GAN11, AKAP9 and SMC4 were identified as driver genes in the specimens of 2 patients. In this small cohort, we also found 9 mutated genes, including TP53, TNS4, ZNF208, ZNF254, PRG4, KRTAP21-1, FOXD4L1, SLC38A9 and ZNF117. Whether these represented significantly recurrent or driver mutations in high-grade LG MEC remains undetermined and requires statistical validation in larger patient series.

## Discussion

This study enrolled clinical data from two well-recognized tertiary ophthalmic and orbital disease centers in China over a 14-year period, establishing a relatively standardized registry system for case recruitment, longitudinal follow-up, and pathological archiving of lacrimal gland tumours. In this study, we have, for the first time, conducted a comprehensive analysis of the clinical manifestations, imaging characteristics, treatment outcomes, pathological features and mutational landscape for the highly malignant and lethal primary high-grade LG MEC. To enhance our understanding of this rare condition, we further conducted an exhaustive review and synthesis of previously documented cases of LG MECs with clear pathological grading, as presented in Supplementary Table S5.

Based on our series of 20 cases and 21 cases from the literatures (Supplementary Table S5), high-grade LG MEC predominantly affects elderly males, with a male-to-female ratio of 1.9:1, and primarily presents as painless exophthalmos. Although painless exophthalmos is not a specific clinical manifestation of LG MEC, it serves as a crucial distinguishing feature from LG ACC. Because both our findings and existing literature emphasize high occurrences of ocular pain in LG ACC patients (50%~80%), [12] in addition to symptoms such as exophthalmos and ocular dyskinesia.

We, for the first time, found that the characteristic radiologic findings of primary high-grade LG MEC included calcification and bone destruction, hypointense on T2WI and non-enhanced areas on contrast-enhanced T1WI. These MRI characteristics were consistent with histopathological features such as few mucous cells, limited mucous secretion, abundant collagen deposition, and a predilection for coagulative necrosis in primary high-grade LG MEC, providing a potential explanation for these distinct imaging features. In contrast, LG ACC has a lower incidence of calcification and typically presents as isointense or hyperintense on T2WI, with fewer instances of non-enhancing areas on contrast-enhanced T1WI [13, 14].

Complete surgical resection combined with adjuvant radiotherapy is currently the mainstay treatment of LG malignancies [15]. Although adjuvant therapy significantly improved recurrence‑free survival in patients with primary high-grade LG MEC undergoing complete resection, it did not translate into a meaningful benefit in overall survival, indicating that adjuvant radiotherapy yields limited efficacy for prolonging long‑term survival. Several factors may account for these findings. First, the relatively small sample size and imbalanced distribution across treatment groups reduced the statistical power of the overall survival analysis. The Log-rank test for trend yielded a *P* value close to 0.05, indicating a potential linear prognostic gradient that failed to reach significance owing to limited cohort size; accordingly, validation in larger patient series is required. Second, the primary high-grade LG MEC exhibits aggressive biological behavior and high malignant potential. Despite comprehensive postoperative management, advanced disease remains prone to distant metastasis, which contributes substantially to mortality and counteracts the local therapeutic benefits achieved by adjuvant intervention. Our cohort of 20 primary high-grade LG MEC patients exhibited recurrence, metastasis, and mortality rates of 52.9%, 71.4%, and 85%, respectively. In comparison, existing literature on 21 cases of primary high-grade LG MEC report rates of 27.8% (5/18), 36.8% (7/19), and 38.1% (8/21) (Supplementary Table S5). The observed disparity in these rates may be attributed to our longer follow-up period of 35.5 months compared to the literature’s 12 months. Overall, the mortality rate among 41 primary high-grade LG MEC cases could potentially exceed 61%, surpassing the mortality rates of other ocular diseases with high fatality rates such as LG ACC (22%-50%) [16] and uveal melanoma (~ 40%) [17]. We therefore propose that primary high-grade LG MEC may be the ocular disease with the highest mortality rate. In comparison, other types of LG MEC generally show much more favorable prognoses. Our study, along with existing literature (Supplementary Table S5), demonstrated that medium-grade LG MEC cases treated with complete excision, with or without radiotherapy, did not experience recurrence, metastasis, or mortality. Furthermore, literature findings indicate that among 17 cases of low-grade LG MEC, recurrence, metastasis, and mortality rates are 0%, 5.9%, and 6.7%, respectively (Supplementary Table S5).

For the first time, we found that TNM staging was a key prognostic risk factor in patients with high-grade LG MEC and patients in stage T4 carried the worst prognosis. Specifically, among the 12 patients with T4 primary high-grade LG MEC in our cohort, the rates of recurrence, metastasis, and mortality were 77.8%, 71.4%, and 100%, respectively. In the previous studies, 5 patients with T4 primary high-grade LG MEC exhibited recurrence, metastasis, and mortality rates were 66.7%, 75%, and 80%, respectively, over a mean follow-up period of 12.4 months (Supplementary Table S5). In contrast, 8 patients with T1-3 primary high-grade LG MEC and 9 cases reported in the literatures exhibited recurrence, metastasis, and mortality rates of 23.5%, 52.9% and 47.1% (Supplementary Table S5). Therefore, early detection, early diagnosis and timely intervention for T1-3 lesions substantially improved survival outcomes of high-grade LG MEC. Owing to the limited sample size (8 patients in the T1-3 group and 12 in the T4 group), the confidence interval for the median survival ratio was wide, reflecting insufficient statistical power. Further validation in larger patient cohorts was therefore required to confirm these observations.

These high rates of recurrence, metastasis, and mortality highlight the pressing necessity for novel treatment approaches for primary high-grade LG MEC. For the first time, we elucidated the mutational landscape of primary high-grade LG MEC and identified the driver genes and significantly mutated genes. These findings established a crucial foundation for the development of novel therapeutic strategies. Despite the limited data from only 3 patients, each exhibited a missense mutation in the TP53 gene, which was preliminarily identified as both driver gene and significantly mutated gene. Consistently, previous study has suggested that TP53 and POU6F2 mutations may be the main drivers of salivary gland MEC [18]. Specifically, TP53 mutations have been observed exclusively in medium and high-grade salivary gland MEC, whereas POU6F2 mutations have been detected solely in low-grade salivary gland MEC [18]. Furthermore, recent whole-exome sequencing of 26 salivary gland MECs has also demonstrated that TP53 mutations were more frequently found in aggressive tumor phenotypes [19]. These findings suggested that the targeted therapy for TP53 mutations could be a potential therapeutic approach for primary high-grade LG MEC. In addition, we also found that EGFR, DOCK2, GAN11, AKAP9 and SMC4 were identified as driver genes in the specimens of 2 patients. Previous study has also indicated that EGFR gene abnormality could play an important role in the development of high-grade salivary gland MEC, suggesting that molecular targeted therapies against EGFR could hold promise as a treatment option [20]. Three cases of high-grade salivary gland MEC with EGFR overexpression, have successively reported partial radiological response after treatment with cetuximab, an EGFR inhibitor [21–23]. Given the substantial EGFR positivity rate observed in high-grade LG MEC (63.2%), it is hypothesized that EGFR inhibitors may represent promising therapeutic agents for the treatment of this malignancy. The present study has a critical limitation related to the small sample size in the mutational profiling analysis. Although several potential candidate genes were preliminarily identified, rigorous statistical validation could not be achieved. Accordingly, larger-scale sequencing cohorts and functional experimental validation are warranted in future investigations.

## Conclusions

Taken together, primary high-grade LG MEC primarily manifesting as painless exophthalmos. Imaging studies typically reveal irregular, ill-defined LG lesions, often accompanied by calcification and bone destruction, with typically hypointense on T2WI and non-enhanced areas on contrast-enhanced T1WI. Adjuvant radiotherapy after complete resection demonstrated limited therapeutic effect. TNM staging significantly influences the prognosis of patients with high-grade LG MEC and patients in stage T4 carry the worst prognosis. Targeted treatment of TP53 or EGFR is a potential therapeutic approach that needs to be confirmed in future clinical trials.

## Supplementary Information

Below is the link to the electronic supplementary material.

Supplementary Material 1

## Data Availability

The whole exome sequencing data generated during the current study are available in the China National Center for Bioinformation repository, [GSA-Human: HRA010562].
