# Supplementary material for: Primary high-grade mucoepidermoid carcinoma of the lacrimal gland: retrospective clinical-pathologic analysis of 20 cases
Source: BMC Ophthalmol. 2026 Apr 17;26:295. doi: 10.1186/s12886-026-04784-y (PMC13224526; doi:10.1186/s12886-026-04784-y)
Supplement: Supplementary file 1 — Supplementary Material 1 [file 12886_2026_4784_MOESM1_ESM.docx]

**Primary high-grade mucoepidermoid carcinoma of the lacrimal gland: retrospective clinical-pathologic analysis of 20 cases**

**SUPPLEMENTARY MATERIAL**


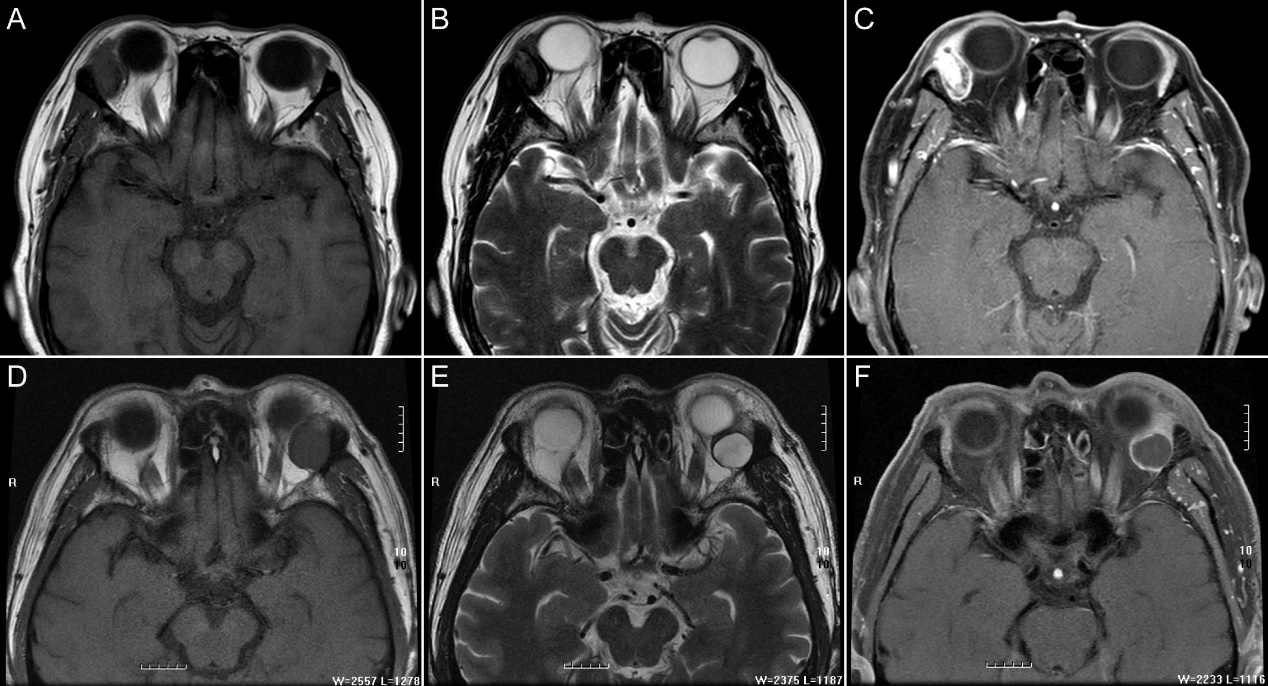


Figure S1. MRI characteristics of medium-grade LG MEC

(A) T1WI of MRI scan showed the medium-grade LG MEC lesion was isointensity. (B) T2WI of MRI scan showed the medium-grade LG MEC lesion was isointensity. (C) Contrast-enhanced T1WI of MRI scan showed intermediate enhancement and peripheral lack of enhancement in the medium-grade LG MEC lesion. The MRI scans in A-C were from the same patient. (D) T1WI of MRI scan showed the medium-grade LG MEC lesion was isointensity. (E) T2WI of MRI scan showed the medium-grade LG MEC lesion was hyperintensity. (F) Contrast-enhanced T1WI of MRI scan showed peripheral enhancement and central lack of enhancement in the medium-grade LG MEC lesion. The MRI scans in D-F were from the same patient.

**Table S1.** Baseline characteristics

|  | High-grade LG MEC (n=20) | LG ACC (n=35) | *P* |
| --- | --- | --- | --- |
| Age, mean, years | 60.50±13.6 | 41.2±15.6 | ＜0.001 |
| Gender |  |  | 0.010 |
| Male | 17 | 17 |  |
| Female | 3 | 18 |  |
| Proptosis | 85% | 71.4% | 0.333 |
| Ocular dyskinesia | 75% | 45.7% | 0.049 |
| Palpable periorbital mass | 65% | 51.4% | 0.403 |
| Eyeball displacement | 60% | 31.4% | 0.05 |
| Swollen eyelid | 60% | 57.1% | 1.000 |
| Ptosis | 30% | 14.3% | 0.181 |
| Visual loss | 20% | 22.9% | 1.000 |
| Ocular pain | 20% | 54.3% | 0.022 |

**Table S2.** Comparison of radiologic features between high-grade LG MEC and LG ACC

|  | High-grade LG MEC | LG ACC | *P* |
| --- | --- | --- | --- |
| Homogeneous mass | 89.5% (17/19) | 81.3% (13/16) | 0.642 |
| Bone destruction | 89.5% (17/19) | 75.0% (12/16) | 0.379 |
| Calcification | 73.7% (14/19) | 18.8% (3/16) | 0.002 |
| Isointense in T1WI | 100.0% (20/20) | 91.3% (21/23) | 0.491 |
| Hypointense in T2WI | 85.0% (17/20) | 13.0% (3/23) | ＜0.001 |
| Non-enhanced areas in contrast-enhanced T1WI | 95.0% (19/20) | 52.2% (12/23) | 0.002 |

**Table S3.** Treatment and prognosis of LG MEC

| Patient  NO. | Age | TNM | Histopathology | Treatment | Recurrence | Metastasis | Survival |
| --- | --- | --- | --- | --- | --- | --- | --- |
| 1 | 73 | T4cN0M0 | high-grade | complete resection + radiotherapy | Recurrence in 21 m | Metastasis in 6 m (Lung) | Dead in 24 m |
| 2 | 68 | T4cN0M0 | high-grade | complete resection + radiotherapy | N/A | Metastasis in 1 m (Bone) | Dead in 7 m |
| 3 | 63 | T4cN0M0 | high-grade | complete resection + radiotherapy | No recurrence within 25 m | Metastasis in 25 m (Lung, bone) | Dead in 53 m |
| 4 | 69 | T4cN0M0 | high-grade | complete resection + radiotherapy | Recurrence in 12 m (extension to brain) | No metastasis within 60 m | Dead in 60 m |
| 5 | 75 | T4cN0M0 | high-grade | complete resection | N/A | N/A | Dead in 42 m |
| 6 | 71 | T4cN0M0 | high-grade | complete resection | N/A | N/A | Dead in 23 m |
| 7 | 60 | T4bN0M0 | High-grade | complete resection + radiotherapy | No recurrence within 6 m | No metastasis within 6 m | Dead in 36 m |
| 8 | 65 | T4cN0M1 | high-grade | subtotal resection + chemotherapy | Recurrence in 13 m (extension to brain) | N/A | Dead in 19 m |
| 9 | 62 | T4bN0M0 | high-grade | subtotal resection + radiotherapy | Recurrence in 54 m (extension to brain) | No metastasis within 63 m | Dead in 63 m |
| 10 | 51 | T4bN0M0 | high-grade | subtotal resection + radiotherapy | Recurrence in 8 m | No metastasis within 24 m | Dead in 24 m |
| 11 | 84 | T4bN0M0 | high-grade | subtotal resection + radiotherapy + lenvatinib | Recurrence in 0.7 m (extension to brain) | Metastasis in 3 m (Bone) | Dead in 4 m |
| 12 | 50 | T4bN0M0 | high-grade | subtotal resection + radiotherapy | Recurrence in 24 m | Metastasis in 6 m (Lung) | Dead in 35 m |
| 13 | 77 | T3cN0M0 | high-grade | complete resection + radiotherapy | No recurrence within 12 m | No metastasis within 12 m | Alive, no evidence of disease within 12 m |
| 14 | 36 | T2cN0M0 | high-grade | complete resection + radiotherapy | No recurrence within 8 m | Metastasis in 8 m (Brain, lung) | Dead in 63 m |
| 15 | 46 | T2cN0M0 | high-grade | complete resection + radiotherapy | Recurrence in 28 m | Metastasis in 28 m (Parotid gland, cervical lymph nodes) | Dead in 49 m |
| 16 | 65 | T2cN0M0 | high-grade | complete resection + radiotherapy | No recurrence within 79 m | Metastasis in 72 m (Lung) | Dead in 79 m |
| 17 | 32 | T2bN0M0 | high-grade | complete resection + radiotherapy | No recurrence within 153 m | No metastasis within 153 m | Alive, no evidence of disease within 153 m |
| 18 | 49 | T2bN0M0 | high-grade | complete resection + radiotherapy | No recurrence within 10 m | Metastasis in 3 m (Lung, parotid gland and lymph nodes) | Dead in 10 m |
| 19 | 51 | T2bN0M0 | high-grade | complete resection | Recurrence in 3 m | Metastasis in 2.5 m (Parotid gland) | Alive, with disease within 88 m |
| 20 | 63 | T2cN1M1 | high-grade | complete resection + chemotherapy | No recurrence within 22 m | Metastasis in 6 m (Lung) | Dead in 22 m |
| 21 | 63 | T2cN0M0 | medium-grade | complete resection | No recurrence within 10 m | No metastasis within 10 m | Alive, no evidence of disease within 10 m |
| 22 | 55 | T1N0M0 | medium-grade | complete resection + radiotherapy | No recurrence within 59 m | No metastasis within 59 m | Alive, no evidence of disease within 59 m |

N/A=not applicable; TNM = AJCC TNM Classification 8th edition criteria

**Supplementary Table S4.** Driver genes identified in 3 high-grade LG MEC cases

|  | Gene Symbol | Nucleotide (genomic) | Mutation Type | IntOGen Cancer | CGC Cancer | Sample ID |
| --- | --- | --- | --- | --- | --- | --- |
| 1 | TP53 | chr17_7675088_C_T | Missense | ACC, ALL, AML, AN, ANGS, BC, BLCA, BLY, BRCA, CESC, CH, CLL, CM, COREAD, DLBCL, ESCA, EWS, GBM, HC, HGG, HNSC, LEIS, LGG, LUAD, LUSC, LY, MBL, MESO, MM, NHLY, NSCLC, NSPH, OS, OV, PAAD, PAIS, PIA, PRAD, RCCC, RCH, RPC, S, SBCC, SCLC, SSCC, ST, THCA, THYM, UCEC, UCS, VV, WT | breast; colorectal; lung; sarcoma; adrenocortical; glioma; Spitzoid Tumor; multiple other Tumor types | 1 |
| 2 | AKAP9 | chr7_92079176_A_G | Missense | N/A | N/A | 1 |
| 3 | SMC4 | chr3_160431815_A_G | Missense | N/A | N/A | 1 |
| 4 | DOCK2 | chr5_ 169864226_C_T | Intron | N/A | N/A | 1 |
| 5 | EGFR | chr7_ 55170357_C_T | Intron | N/A | glioma; NSCLC | 1 |
| 6 | GNA11 | chr19_3110033_C_T | Intron | CM, UVM | uveal melanoma; primary central nervous system melanocytic neoplasms | 1 |
| 7 | ARHGAP35 | chr19_46937109_G_A | Intron | LUSC, UCEC | N/A | 1 |
| 8 | ARID1B | chr6_157148787_G_C | Missense | N/A | breast; hepatocellular carcinoma; clear cell ovarian carcinoma | 1 |
| 9 | CCND3 | chr6_42033185_C_T | Intron | N/A | MM | 1 |
| 10 | CDH1 | chr16_68827019_T_C | Intron | BLCA, BRCA, DLBCL, SSCC, ST | lobular breast; gastric | 1 |
| 11 | CHD3 | chr17_7893623_C_T | Intron | N/A | N/A | 1 |
| 12 | CLIP1 | chr12_122365285_A_G | Intron | N/A | N/A | 1 |
| 13 | DOCK4 | chr7_ 112163281_C_T | Intron | N/A | N/A | 1 |
| 14 | EXT2 | chr11_44124835_G_A | Missense | N/A | N/A | 1 |
| 15 | FRG1B | chr20_30378182_C_T | RNA | N/A | N/A | 1 |
| 16 | GRIN2A | chr16_ 9960869_T_A | Intron | BLCA, BRCA, COREAD, ST | melanoma; colorectal carcinoma; gastric carcinoma; lung carcinoma | 1 |
| 17 | INPP4A | chr2_ 98538090_G_C | Intron | N/A | N/A | 1 |
| 18 | IRF4 | chr6_ 394744_G_T | Intron | CLL, DLBCL, MM, NHLY | MM | 1 |
| 19 | JAK3 | chr19_ 17840021_A_G | Intron | N/A | acute megakaryocytic leukaemia; ETP ALL | 1 |
| 20 | MAST2 | chr1_45948474_A_T | Intron | N/A | N/A | 1 |
| 21 | MED17 | chr11_93807325_C_A | Intron | N/A | N/A | 1 |
| 22 | MEGF6 | chr1_3510088_G_A | Intron | N/A | N/A | 1 |
| 23 | NEK1 | chr4_169567646_G_A | Intron | N/A | N/A | 1 |
| 24 | PBRM1 | chr3_52644652_A_G | Intron | N/A | clear cell renal carcinoma; breast | 1 |
| 25 | POLG | chr15_89321924_C_T | Intron | N/A | CRC | 1 |
| 26 | POTEF | chr2_130102093_T_C | Intron | N/A | N/A | 1 |
| 27 | RANBP3 | chr19_5934549_T_C | Intron | N/A | N/A | 1 |
| 28 | REL | chr2_60905379_C_T | Intron | N/A | Hodgkin lymphoma | 1 |
| 29 | RMI2 | chr16_11350449_G_A | Intron | N/A | PMBL; Hodgkin lymphoma | 1 |
| 30 | SMARCAD1 | chr4_94273811_G_T | Intron | N/A | N/A | 1 |
| 31 | TNKS1BP1 | chr11_57313798_G_C | Nonsense | N/A | N/A | 1 |
| 32 | VEZT | chr12_ 95266538_G_C | Missense | N/A | N/A | 1 |
| 33 | TP53 | chr17_7676076_G_A | Missense | ACC, ALL, AML, AN, ANGS, BC, BLCA, BLY, BRCA, CESC, CH, CLL, CM, COREAD, DLBCL, ESCA, EWS, GBM, HC, HGG, HNSC, LEIS, LGG, LUAD, LUSC, LY, MBL, MESO, MM, NHLY, NSCLC, NSPH, OS, OV, PAAD, PAIS, PIA, PRAD, RCCC, RCH, RPC, S, SBCC, SCLC, SSCC, ST, THCA, THYM, UCEC, UCS, VV, WT | breast; colorectal; lung; sarcoma; adrenocortical; glioma; Spitzoid Tumor; multiple other Tumor types | 2 |
| 34 | SMC4 | chr3_160423883_A_T | Intron | N/A | N/A | 2 |
| 35 | AKAP9 | chr7_92002061_A_G | Missense | N/A | N/A | 2 |
| 36 | BAP1 | chr3_52407219_G_A | Missense | BRCA, CESC, CH, CM, ESCA, HC, MESO, NSCLC, PAAD, RCCC, RPC, UVM | uveal melanoma; breast; NSCLC; RCC | 2 |
| 37 | CFH | chr1_196746038_G_A | Intron | N/A | N/A | 2 |
| 38 | DGCR8 | chr22_ 20086473_C_T | Silent | DLBCL, THCA, WT | Wilms Tumor | 2 |
| 39 | KMT2D | chr12_49048045_A_G | Missense | ACY, ALL, AN, BLCA, BRCA, CESC, CH, CLL, DLBCL, ESCA, GBM, HC, HGG, HNSC, LUSC, LY, MBL, MESO, NB, NHLY, NSCLC, OV, PAAD, PIA, PRAD, RCH, SBCC, SCLC, SSCC, ST, UCEC | medulloblastoma; renal | 2 |
| 40 | LRP1B | chr2_141247222_T_G | Intron | AML, AN, BRCA, CH, CLL, CM, COREAD, ESCA, GBM, HC, HNSC, LUAD, LUSC, MBL, MGCT, MM, NB, NSCLC, OS, OV, PAAD, PRAD, RCCC, RHBDS, SBCC, SCLC, ST, THCA, UCS, UVM | CLL; ovarian cancer; oesophageal squamous cell carcinoma; urothelial cancer | 2 |
| 41 | SETD1B | chr12_121825349_C_A | Missense | ESCA, OV, PAAD | CRC; endometrium | 2 |
| 42 | SPATA6 | chr1_48436530_C_T | Intron | N/A | N/A | 2 |
| 43 | SPEG | chr2_219464452_G_A | Missense | N/A | N/A | 2 |
| 44 | TP53 | chr17_ 7674885_C_T | Missense | ACC, ALL, AML, AN, ANGS, BC, BLCA, BLY, BRCA, CESC, CH, CLL, CM, COREAD, DLBCL, ESCA, EWS, GBM, HC, HGG, HNSC, LEIS, LGG, LUAD, LUSC, LY, MBL, MESO, MM, NHLY, NSCLC, NSPH, OS, OV, PAAD, PAIS, PIA, PRAD, RCCC, RCH, RPC, S, SBCC, SCLC, SSCC, ST, THCA, THYM, UCEC, UCS, VV, WT | breast; colorectal; lung; sarcoma; adrenocortical; glioma; Spitzoid Tumor; multiple other Tumor types | 3 |
| 45 | DOCK2 | chr5_ 170079999_G_A | Intron | N/A | N/A | 3 |
| 46 | EGFR | chr7_ 55202873_C_T | 3'UTR | N/A | glioma; NSCLC | 3 |
| 47 | GNA11 | chr19_ 3114968_C_A | Silent | CM, UVM | uveal melanoma; primary central nervous system melanocytic neoplasms | 3 |
| 48 | EGR3 | chr8_ 22690637_C_A | Missense | N/A | N/A | 3 |
| 49 | ABI1 | chr10_ 26839710_G_T | Intron | N/A | AML | 3 |
| 50 | ACVR1 | chr2_157753325_T_C | Intron | HGG, UCEC | DIPG | 3 |
| 51 | AMPH | chr7_38461465_C_T | Intron | N/A | N/A | 3 |
| 52 | ARHGAP32 | chr11_128985910_C_A | Intron | N/A | N/A | 3 |
| 53 | ARHGEF6 | chrX_136685660_G_A | Intron | N/A | N/A | 3 |
| 54 | ARID1A | chr1_26721482_G_A | Intron | N/A | clear cell ovarian carcinoma; RCC; breast | 3 |
| 55 | ATM | chr11_ 108257659_A_G | Intron | N/A | T-PLL | 3 |
| 56 | CACNA1D | chr3_53779037_A_G | Intron | N/A | adrenal aldosterone producing adenoma | 3 |
| 57 | CAMTA1 | chr1_6830312_A_G | Intron | N/A | Epithelioid haemangioendothelioma | 3 |
| 58 | CDKN1A | chr6_36684236_C_A | Silent | N/A | bladder cancer | 3 |
| 59 | CPEB3 | chr10_92227870_C_A | Intron | N/A | lung cancer | 3 |
| 60 | CREBBP | chr16_3728875_G_A | Missense | ALL, BLCA, BRCA, CLL, DLBCL, ESCA, HC, HNSC, LUSC, LY, MBL, NHLY, PAIS, PIA, SBCC, SCLC, SG, VV | ALL; AML; DLBCL; B-NHL | 3 |
| 61 | DDX5 | chr17_ 64501415_C_A | Intron | N/A | prostate | 3 |
| 62 | DNM3 | chr1_ 172339182_A_G | Intron | N/A | N/A | 3 |
| 63 | EIF3E | chr8_ 108239408_C_G | Intron | CESC, COREAD | colorectal | 3 |
| 64 | ETV5 | chr3_ 186082666_A_G | Intron | AML, BRCA | prostate | 3 |
| 65 | EWSR1 | chr22_ 29286166_G_A | Intron | EWS | Ewing sarcoma; desmoplastic small round cell Tumor ; ALL; clear cell sarcoma; sarcoma; myoepithelioma; mesothelioma | 3 |
| 66 | FES | chr15_90890064_C_T | Intron | N/A | HNSCC; ovarian carcinoma | 3 |
| 67 | FGFR3 | chr4_1806863_C_A | Missense | N/A | bladder; MM; T-cell lymphoma | 3 |
| 68 | FGFR4 | chr5_177091724_G_A | Missense | GBM | rhabdomyosarcoma | 3 |
| 69 | FLNA | ChrX_154359088_C_T | Missense | N/A | N/A | 3 |
| 70 | FOXO3 | chr6_108567784_T_C | Intron | N/A | AL | 3 |
| 71 | HIP1 | chr7_ 75616572_T_C | Intron | N/A | CMML; NSCLC | 3 |
| 72 | IGF2BP2 | chr3_ 185719632_C_A | Intron | N/A | lymphoma | 3 |
| 73 | KDM6A | chrX_ 44873854_C_T | Intron | ALL, BLCA, BRCA, CH, ESCA, HNSC, LUSC, MBL, PAAD, PIA, PRAD, RPC, SCLC, SSCC, ST | renal cell carcinoma; bladder carcinoma; oesophageal SCC; MM; medulloblastoma; T-ALL; other Tumor types | 3 |
| 74 | KIF20B | chr10_89738065_G_A | Missense | N/A | N/A | 3 |
| 75 | MACF1 | chr1_39386825_C_A | Intron | N/A | N/A | 3 |
| 76 | MAP3K13 | chr3_185291576_G_C | Intron | N/A | breast | 3 |
| 77 | MAP4 | chr3_47930183_C_T | Intron | N/A | N/A | 3 |
| 78 | MED12 | ChrX_71121240_G_T | Intron | CESC, CLL, GBM, LGG, NB, OS, PRAD | uterine leiomyoma; fibroadenoma; phyllodes Tumor | 3 |
| 79 | MLLT10 | chr10_21642118_A_G | Intron | N/A | AL | 3 |
| 80 | MUC16 | chr19_8892619_G_A | Silent | N/A | HNSCC; melanoma | 3 |
| 81 | NAV1 | chr1_201733089_G_A | Intron | N/A | N/A | 3 |
| 82 | NBEA | chr13_35668428_G_A | Missense | N/A | N/A | 3 |
| 83 | NCOA2 | chr8_70137263_A_G | Intron | LUAD, LUSC | AML; chondrosarcoma; rhabdomyosarcoma | 3 |
| 84 | NF1 | chr17_31359738_A_G | Intron | N/A | neurofibroma; glioma | 3 |
| 85 | NSD2 | chr4_1955194_C_A | Missense | ALL | MM | 3 |
| 86 | NTRK1 | chr1_156875672_G_A | Intron | BRCA | papillary thyroid; Spitzoid Tumor | 3 |
| 87 | NUP214 | chr9_131135251_T_A | Intron | BCNET, MM, RPC | N/A | 3 |
| 88 | PER1 | chr17_8146267_G_A | Intron | N/A | AML; CMML | 3 |
| 89 | RAD51B | chr14_68499787_G_T | Intron | N/A | lipoma; uterine leiomyoma | 3 |
| 90 | RAF1 | chr3_12666875_C_T | 5'Flank | BLCA, CM | pilocytic astrocytoma; prostate | 3 |
| 91 | RALGAPA1 | chr14_35575857_G_T | Intron | N/A | N/A | 3 |
| 92 | RARA | chr17_40354552_C_T | Intron | N/A | APL | 3 |
| 93 | RBM10 | ChrX_47173151_C_T | Silent | N/A | lung adenocarcinoma | 3 |
| 94 | RET | chr10_43106631_C_A | Intron | CM, LUAD, PCPG, S, THCA | medullary thyroid; papillary thyroid; pheochromocytoma; NSCLC; Spitzoid Tumor | 3 |
| 95 | ROBO2 | chr3_77522851_G_C | Missense | N/A | colorectal adenocarcinoma; melanoma | 3 |
| 96 | RSRC1 | chr3_158149057_T_C | Intron | N/A | N/A | 3 |
| 97 | SF3B1 | chr2_197430736_A_G | Intron | AML, BLCA, BRCA, CH, CLL, CM, LNET, MBL, PAAD, PRAD, RCCC, THYM, UVM | myelodysplastic syndrome | 3 |
| 98 | SGK1 | chr6_134255137_G_A | Intron | DLBCL, LY, NHLY | Nodular lymphocyte predominant Hodgkin lymphoma | 3 |
| 99 | SKI | chr1_2228997_C_A | Silent | N/A | melanoma | 3 |
| 100 | SRCAP | chr16_30724730_C_T | Missense | N/A | N/A | 3 |
| 101 | STAG2 | ChrX_124061792_G_A | Missense | N/A | bladder carcinoma; glioblastoma; melanoma; Ewing's sarcoma; myeloid neoplasms | 3 |
| 102 | STAT5B | chr17_42218654_C_T | Intron | OV | large granular lymphocytic leukaemia; skin basal cell; APL | 3 |
| 103 | SYNE1 | chr6_152148380_T_C | Splice_Site | N/A | N/A | 3 |
| 104 | TFE3 | ChrX_49030233_C_A | Silent | N/A | papillary renal; alveolar soft part sarcoma; renal | 3 |
| 105 | TNC | chr9_115035290_C_A | Nonsense | N/A | SCC; melanoma | 3 |
| 106 | TRIO | chr5_14405666_G_T | Intron | N/A | N/A | 3 |
| 107 | USP8 | chr15_ 50489810_G_A | Missense | N/A | corticotroph adenoma | 3 |
| 108 | VHL | chr3_ 10144537_C_A | Intron | RCCC, THYM | renal; haemangioma; pheochromocytoma | 3 |
| 109 | WNK2 | chr9_ 93251318_T_C | Intron | COREAD, HC, THCA | gastric cancer | 3 |
| 110 | ZFP36L1 | chr14_ 68790016_G_T | 3'UTR | N/A | N/A | 3 |
| 111 | ZMYM3 | chrX_71250296_C_T | Intron | N/A | CRC; breast cancer | 3 |

IntOGen: 568 driver genes identified in pan-cancer data; IntOGen Cancer: Tumor type from IntOGen; CGC: 558 driver genes included in the Cancer Gene Census database; CGC Cancer: Tumor Types from CGC.

**Table S5.** Review of reported cases of LG MEC with clear pathological grading

| Pt. no. | General characteristics | | Radiological images | | Histopathology | TNM | Management | Prognosis | | |
| --- | --- | --- | --- | --- | --- | --- | --- | --- | --- | --- |
|  | Age/  sex | Symptoms | CT | MRI |  |  |  | Recurrence | Metastasis | Survival |
| 1^1^ | 52/M | painless, swollen eyelid, palpable mass, restricted ocular motility | N/A | T1 isointense, T2 hyperintense, heterogeneous contrast enhancement | high-grade | T4cN0M0 | marginal excision + radiotherapy | Recurrence in 10 m (3 times) | Metastasis in 10 m (submandibular gland nodule firstly) | Dead of disease after 20 m |
| 2^2^ | 72/F | proptosis, painless, palpable mass, diplopia | well-circumscribed, calcification | N/A | intermediate to high-grade mucoepidermoid CEPA | T2aN0M0 | marginal excision + radiotherapy | No recurrence within 5 m | No metastasis within 5 m | Alive, no evidence of disease within 5 m |
| 3^3^ | 55/F | proptosis, painful, swollen eyelid, palpable mass, eyeball displacement, restricted ocular motility | well-circumscribed, bony remodeling | T2 hyperintense | high-grade mucoepidermoid CEPA | T1aN0M0 | marginal excision + radiotherapy | No recurrence within 12 m | No metastasis within 12 m | Alive, no evidence of disease within 12 m |
| 4^4^ | 67/M | proptosis, painless, palpable mass, restricted eye movements, decreased vision, epiphora | N/A | N/A | high-grade | T2cNxMx | orbital exenteration + radiotherapy + chemotherapy | Recurrence within 6 m | Metastasis within 6 m (Lung) | Dead of disease after 6 m |
| 5^4^ | 50/F | painless, palpable mass | N/A | N/A | high-grade | T2xNxMx | marginal excision + radiotherapy | No recurrence within 10 m | Metastasis within 10 m (brain) | Dead of disease after 10 m |
| 6^4^ | 72/F | painless | N/A | N/A | high-grade | T1xNxMx | marginal excision + radiotherapy | No recurrence within 11 m | No metastasis within 11 m | Alive, no evidence of disease within 11 m |
| 7^5^ | 60/M | proptosis, painful, palpable mass, eyeball displacement, restricted ocular motility, epiphora, decreased vision | well-defined | N/A | high-grade | T2xN0M0 | marginal excision | No recurrence within 15 m | No metastasis within 15 m | Alive, no evidence of disease within 15 m |
| 8^6^ | 45/M | painful, palpable mass, decreased vision (nil light perception) | N/A | N/A | high-grade | N/A | orbital exenteration + incomplete radiotherapy | No recurrence within 12m | Metastasis within 12 m | Dead of disease after 12 m |
| 9^6^ | 32/F | palpable mass, decreased vision (counting finger) | ill-defined, calcification, bony remodeling | N/A | high-grade | N/A | marginal excision | No recurrence within 6 m | No metastasis within 6 m | Alive, no evidence of disease within 6 m |
| 10^7^ | 79/F | proptosis, painless, swollen eyelid, ptosis, palpable mass, eyeball displacement | ill-defined, bony destruction | heterogeneous contrast enhancement | high-grade | T4cN0M0 | marginal excision + radiotherapy | No recurrence within 13 m | Metastasis within 13 m (parotid gland) | Dead of disease after 26 m |
| 11^8^ | 62/F | proptosis, painful, palpable mass, eyeball displacement | ill-defined, calcification | N/A | high-grade mucoepidermoid CEPA | T2aN0M0 | marginal excision | Recurrence within 30 m | No metastasis within 30 m | Alive with recurrent disease within 30 m |
| 12^9^ | 55/M | N/A | N/A | N/A | high-grade | N/A | orbital exenteration + radiotherapy | Recurrence within 144 m | No metastasis within 144 m | Alive with recurrent disease within 144 m |
| 13^9^ | 49/M | N/A | N/A | N/A | high-grade | TxNxM1 | orbital exenteration + chemotherapy | No recurrence | No metastasis | Alive, no evidence of disease |
| 14^10^ | 76/F | proptosis, palpable mass, eyeball displacement, restricted ocular motility | ill-defined, calcification, defect in orbital roof | N/A | high-grade | T4cN0M0 | marginal excision + radiotherapy + chemotherapy | Recurrence in 1 m | No metastasis within 9 m | Alive with recurrent disease within 9 m |
| 15^10^ | 53/M | proptosis, painless, palpable mass, eyeball displacement | irregular | N/A | high-grade | T2xN0M0 | marginal excision | No recurrence within 13 m | Metastasis within 6 m (parotid gland, lymph node and lung) | Dead of disease after 13 m |
| 16^10^ | 79/M | proptosis, swollen eyelid, eyeball displacement, restricted ocular motility, diplopia | bony destruction | N/A | high-grade | T4xNxM1 | radiotherapy | N/A | Metastasis within 6 m (claviculate) | Dead of disease in 6 m after initial examination |
| 17^10^ | 37/M | proptosis, painful, swollen eyelid, | irregular | N/A | high-grade | T1cN0M0 | orbital exenteration + radiotherapy | No recurrence within 17 m | No metastasis within 17 m | Alive, no evidence of disease within 17 m |
| 18^10^ | 33/F | N/A | N/A | N/A | high-grade | N/A | orbital exenteration + radiotherapy | N/A | N/A | Alive, dead of disease within 39 |
| 19^10^ | 29/F | proptosis | N/A | N/A | high-grade | N/A | marginal excision | No recurrence within 6 m | No metastasis within 6 m | Alive, no evidence of disease within 6 m |
| 20^11^ | 62/M | proptosis, painful, swollen eyelid, ptosis | ill-defined, bony destruction | N/A | high-grade | T4cNxMx | nil | N/A | N/A | Dead of disease after 1 m |
| 21^12^ | 72/F | N/A | N/A | N/A | high-grade | N/A | orbital exenteration + radiotherapy | No recurrence within 48 m | No metastasis within 48 m | Alive, no evidence of disease within 48 m |
| 22^13^ | 62/F | proptosis, painless, palpable mass, restricted ocular motility, tearing, photophobia | well-defined, calcification, bony remodeling | N/A | intermediate-grade | T2xN0M0 | marginal excision + radiotherapy | No recurrence within 5 m | No metastasis within 5 m | Alive, no evidence of disease within 5 m |
| 23^4^ | 56/F | proptosis, painless, palpable mass, eyeball displacement, decreased vision | N/A | N/A | low-grade | N/A | marginal excision | No recurrence within 45 m | No metastasis within 45 m | Alive, no evidence of disease within 45 m |
| 24^4^ | 18/F | proptosis, painless, palpable mass | N/A | N/A | low-grade | T2aN0M0 | marginal excision + radiotherapy | No recurrence within 56 m | No metastasis within 56 m | Alive and well within 56 m |
| 25^4^ | 55/F | painless, palpable mass | N/A | N/A | low-grade | T1aN0M0 | marginal excision | No recurrence within 1 m | No metastasis within 1 m | Alive and well within 1 m |
| 26^14^ | 47/F | proptosis, painless, swollen eyelid, palpable mass | well-circumscribed, bony remodeling | T1 isointense, T2 mixed intensity, strong enhancement | low-grade | TxN0M0 | marginal excision | No recurrence | No metastasis | Alive, no evidence of disease |
| 27^6^ | 38/F | proptosis, painful, palpable mass | N/A | N/A | low-grade | N/A | marginal excision | No recurrence within 60 m | No metastasis within 60 m | Alive, no evidence of disease within 60 m |
| 28^6^ | 18/M | palpable mass | N/A | N/A | low-grade | N/A | marginal excision | No recurrence within 60 m | No metastasis within 60 m | Alive, no evidence of disease within 60 m |
| 29^6^ | 17/F | palpable mass | N/A | N/A | low-grade | N/A | marginal excision + radiotherapy | No recurrence within 60 m | No metastasis within 60 m | Alive, no evidence of disease within 60 m |
| 30^15^ | 73/M | painful, palpable mass, swollen eyelid, | well-circumscribed | N/A | low-grade | T1aN0M0 | marginal excision | No recurrence within 60 m | No metastasis within 60 m | Dead of another cause after 60 m |
| 31^16^ | 15/M | painless, palpable mass, eyeball displacement, diplopia | irregular | T1 hypointense, T2 hyperintense, heterogeneous contrast enhancement | low-grade | T2aN0M0 | marginal excision | No recurrence within 24 m | No metastasis within 24 m | Alive, no evidence of disease within 24 m |
| 32^13^ | 70/M | proptosis, painful, swollen eyelid, eyeball displacement, decreased vision | ill-defined, irregular, bony destruction | N/A | low-grade | T4N1M0 | orbital exenteration + radiotherapy + chemotherapy | N/A | Metastasis within 24 m (brain) | Dead of disease after 24 m |
| 33^13^ | 58/F | proptosis, painful, swollen eyelid, eyeball displacement, decreased vision | N/A | N/A | low-grade | T3aN0M0 | marginal excision + radiotherapy | No recurrence within 60 m | No metastasis within 60 m | Alive, no evidence of disease within 60 m |
| 34^10^ | 41/F | N/A | N/A | N/A | low-grade | N/A | marginal excision | No recurrence within 120 m | No metastasis within 120 m | Alive, no evidence of disease within 120 m |
| 35^10^ | 28/M | N/A | N/A | N/A | low-grade | N/A | marginal excision | No recurrence within 24 m | No metastasis within 24 m | Alive, no evidence of disease within 24 m |
| 36^10^ | 62/F | N/A | N/A | N/A | low-grade | N/A | marginal excision | No recurrence within 252 m | No metastasis within 252 m | Dead of another cause after 252 m |
| 37^17^ | 12/F | proptosis, painless, swollen eyelid, ptosis, palpable mass, eyeball displacement, diplopia, decreased vision | well-circumscribed | N/A | low-grade | N/A | marginal excision | No recurrence within 6 m | No metastasis within 6 m | Alive, no evidence of disease within 6 m |
| 38^12^ | 32/M | proptosis | N/A | N/A | low-grade | N/A | orbital exenteration | No recurrence within 12 m | No metastasis within 12 m | Alive, no evidence of disease within 12 m |
| 39^12^ | 72/F | proptosis | N/A | N/A | low-grade | N/A | orbital exenteration | No recurrence within 48 m | No metastasis within 48 m | Alive, no evidence of disease within 48 m |
| 40^18^ | 73/M | painless, swollen eyelid, palpable mass | well-defined, ovoid, bony remodeling | N/A | low-grade | T1N0M0 | marginal excision | No recurrence within 60 m | No metastasis within 60 m | Dead of another cause after 60 m |

**References**

1. Wu Y, He W. Lacrimal gland mucoepidermoid carcinoma with contralateral eye and systemic metastasis: A rare case report and review of the literature. *Front Oncol*. 2023;13:1131587.

2. Topilow NJ, Stevens SM, Chen Y, et al. A rare case of mucoepidermoid carcinoma ex pleomorphic adenoma of the lacrimal gland. *Orbit*. 2023;42(3):311-315.

3. Khademi B, Zia Z, Kohandel-Shirazi M. Mucoepidermoid carcinoma ex pleomorphic adenoma of lacrimal gland: case report and review of literature. *Ophthalmic Plast Reconstr Surg*. 2023;39(1):e22-e25.

4. Sun H, Cai R, Zhai C, et al. Primary mucoepidermoid carcinoma of the lacrimal apparatus. *Am J Ophthalmol*. 2022;239:170-179.

5. Alfin RJ, Alada JJ, Okwudire-Ejeh I, et al. Mucoepidermoid carcinoma of the lacrimal gland: A case report. *J West Afr Coll Surg*. 2020;10(1):26-29.

6. Fasina O, Adeoye AO, Aremu OO. Epithelial lacrimal gland tumours in Nigeria: clinicopathological features and treatment. *J West Afr Coll Surg*. 2020;10(2):5-11.

7. Hwang SJ, Kim KH. High-grade mucoepidermoid carcinoma of the lacrimal gland. *Korean J Ophthalmol*. 2018;32(5):426-427.

8. Daniel L, Rao S, Muthusamy R, et al. Mucoepidermoid carcinoma ex pleomorphic adenoma of the lacrimal gland: a rare presentation. *Indian J Ophthalmol*. 2014;62(6):743-746.

9. Paulino AF, Huvos AG. Epithelial tumors of the lacrimal glands: a clinicopathologic study. *Ann Diagn Pathol*. 1999;3(4):199-204.

10. Eviatar JA, Hornblass A. Mucoepidermoid carcinoma of the lacrimal gland: 25 cases and a review and update of the literature. *Ophthalmic Plast Reconstr Surg*. 1993;9(3):170-181.

11. Lawton AW, Karesh JW. Mucoepidermoid carcinoma of the lacrimal gland fossa: confirmation by ultrastructural study. *South Med J*. 1989;82(5):643-646.

12. Wagoner MD, Chuo N, Gonder JR, et al. Albert DM. Mucoepidermoid carcinoma of the lacrimal gland. *Ann Ophthalmol*. 1982;14(4):383-386.

13. Santos RR, Damasceno RW, de Pontes FS, et al. Ten-year follow-up of a case series of primary epithelial neoplasms of the lacrimal gland: clinical features, surgical treatment and histopathological findings. *Arq Bras Oftalmol*. 2010;73(1):33-39.

14. Makino K, Nakajima K, Tsutsumi S, et al. Mucoepidermoid carcinoma of the lacrimal gland in a patient with the CRTC1-MAML2 fusion gene. *Radiol Case Rep*. 2021;16(12):3643-3646.

15. Von Holstein SL, Fehr A, Heegaard S, et al. CRTC1-MAML2 gene fusion in mucoepidermoid carcinoma of the lacrimal gland. *Oncol Rep*. 2012;27(5):1413-1416.

16. Gedar Totuk OM, Demir MK, Yapicier O, et al. Low-Grade Mucoepidermoid Carcinoma of the Lacrimal Gland in a Teenaged Patient. *Case Rep Ophthalmol Med*. 2017;2017:2418505.

17. Sofinski SJ, Brown BZ, Rao N, et al. Mucoepidermoid carcinoma of the lacrimal gland. Case report and review of the literature. *Ophthalmic Plast Reconstr Surg*. 1986;2(3):147-151.

18. Von Holstein SL. Tumours of the lacrimal gland. Epidemiological, clinical and genetic characteristics. *Acta Ophthalmol*. 2013;91 Thesis 6:1-28.
